# Neural correlates and predictors of subjective cognitive decline in patients with Parkinson’s disease

**DOI:** 10.1007/s10072-021-05734-w

**Published:** 2021-11-25

**Authors:** Anja Ophey, Fabian Krohm, Elke Kalbe, Andrea Greuel, Alexander Drzezga, Marc Tittgemeyer, Lars Timmermann, Frank Jessen, Carsten Eggers, Franziska Maier

**Affiliations:** 1 Department of Medical Psychology | Neuropsychology & Gender Studies, Center for Neuropsychological Diagnostic and Intervention (CeNDI), Faculty of Medicine and University Hospital Cologne, University of Cologne, Kerpener Str. 68, 50937 Cologne, Germany; 2 Department of Neurology, University Hospital of Marburg, Marburg, Germany; 3 Department of Nuclear Medicine, Faculty of Medicine and University Hospital Cologne, University of Cologne, Cologne, Germany; 4 German Center for Neurodegenerative Diseases (DZNE), Bonn-Cologne, Germany; 5 Institute of Neuroscience and Medicine (INM-2), Molecular Organization of the Brain, Forschungszentrum Jülich, Jülich, Germany; 6 Max Planck Institute for Metabolism Research, Cologne, Germany; 7 Excellence Cluster On Cellular Stress Responses in Aging-Associated Diseases (CECAD), University of Cologne, Cologne, Germany; 8 Center for Mind, Brain and Behavior - CMBB, Universities of Marburg and Gießen, Marburg, Germany; 9 Department of Psychiatry and Psychotherapy, Faculty of Medicine and University Hospital Cologne, University of Cologne, Cologne, Germany

**Keywords:** Parkinson disease, Subjective cognitive decline, Neural correlates, Positron emission tomography, Predictors

## Abstract

**Background:**

Subjective cognitive decline (SCD) may occur very early in the course of Parkinson’s disease (PD) before the onset of objective cognitive decline. Data on neural correlates and determinants of SCD in PD are rare.

**Objective:**

The aim of the present study was to identify neural correlates as well as sociodemographic, clinical, and neuropsychological predictors of SCD in patients with PD.

**Methods:**

We retrospectively analyzed 30 patients with PD without cognitive impairment (23% female, 66.90 ± 7.20 years, UPDRS-III: 19.83 ± 9.29), of which *n* = 12 patients were classified as having no SCD (control group, PD-CG) and *n* = 18 as having SCD (PD-SCD). Neuropsychological testing and 18-fluoro-2-deoxyglucose positron emission tomography (FDG-PET) were conducted. SCD was assessed using a questionnaire covering multiple cognitive domains.

**Results:**

SCD subscores differed significantly between PD-CG and PD-SCD and correlated significantly with other scales measuring related concepts. FDG-PET whole-brain voxel-wise regression analysis revealed hypometabolism in middle frontal, middle temporal, and occipital areas, and the angular gyrus as neural correlates of SCD in PD. Next to this hypometabolism, depressive symptoms were an independent significant determinant of SCD in a stepwise regression analysis (adjusted *R*^*2*^ = 50.3%).

**Conclusion:**

This study strengthens the hypothesis of SCD being an early manifestation of future cognitive decline in PD and, more generally, early pathological changes in PD. The early identification of the vulnerability for future cognitive decline constitutes the basis for successful prevention and delay of this non-motor symptom.

**Supplementary Information:**

The online version contains supplementary material available at 10.1007/s10072-021-05734-w.

## Introduction

Subjective cognitive decline (SCD) describes a self-perceived, subjective deterioration in potentially different cognitive domains in the absence of objective cognitive impairment [1]. Subjective deterioration in the memory domain is being discussed to occur at late preclinical stage in Alzheimer’s disease (AD) before the onset of mild cognitive impairment (MCI) and dementia [AD; 1]. SCD, especially in the memory domain, was identified as a risk factor for developing objective cognitive impairment [2, 3] and accelerates time to conversion to objective cognitive impairment [3]. Even though the construct of SCD gained increasing interest in the past decade, heterogeneous instruments predominantly focusing on the memory domain are adopted to assess SCD [4], and recommendations on SCD research criteria in the AD context just emerged [5].

Cognitive decline is a common non-motor symptom in Parkinson’s disease (PD) [6]. Similarly to patients with AD, SCD might constitute a possible precursor of objective cognitive decline in patients with PD [7]. For example, longitudinal studies show significantly higher incidences of PD-MCI and PD dementia for patients with PD and SCD at baseline compared to patients without SCD at baseline [8–10]. In the context of PD, it seems especially reasonable to focus on a broader construct of SCD, as cognitive decline in PD is highly heterogeneous: executive functions, working memory, and attentional functions are among the earliest and most frequently impaired cognitive domains, but memory, visuo-cognitive, and language impairments are observed as well [11–14]. Therefore, the SCD assessment in PD should be based on a broad assessment of cognitive complaints across various cognitive domains. So far, SCD in PD is assessed by a broad range of measures, and there is little consistency between SCD operationalizations [7].

To gain a full understanding of SCD in PD, research on neural correlates and sociodemographic, clinical, and neuropsychological determinants and predictors of SCD is essential. These determinants, as well as neural correlates of SCD, however, have rarely been investigated in PD, and existing studies focused on SCD in the memory domain only. Most of the studies investigating neural correlates of SCD in PD so far categorized their patients with one single question focusing on memory complaints [e.g., “Do you have any memory-related problems?”, 15] and did not use validated SCD assessments.

In normal aging, especially associations of SCD with depressive symptoms and an interplay with objective cognitive functioning have been demonstrated [16]. Furthermore, possible determinants of SCD include demographic variables (e.g., female sex, older age), specific objective cognitive functions (e.g., global cognition, executive functions, language, processing speed), psychological factors (e.g., stress, anxiety), and personality characteristics (e.g., neuroticism) [16–20]. For PD, only anxiety has been identified as a highly significant and independent determinant of SCD so far, whereas age, disease severity, and functioning in instrumental activities of daily living did correspond with SCD [21].

Neural correlates of SCD in the AD context were mainly found in fronto-temporal, temporo-medial, and occipital regions [e.g., 22–25]. Considering morphometric neural correlates of SCD in PD assessed with MRI, cortical thinning and decreased grey matter density was found in the dorsolateral prefrontal, orbitofrontal, medial frontal, and parahippocampal cortices, anterior cingulate, and angular gyrus in patients with PD and SCD in the memory domain compared to PD patients without SCD [9, 15]. Those findings are underlined by a SPECT study [26] demonstrating decreased perfusion in the thalamus and the anterior cingulate as well as in medial frontal and inferior temporal regions.

The objective of the current study was to identify sociodemographic, clinical, and neuropsychological determinants as well as functional neural correlates of SCD in patients with PD assessed with 18-fluoro-2-deoxyglucose positron emission tomography (FDG-PET) which have, to the authors’ best knowledge, not been investigated in combination so far.

## Methods

### Study sample

For this study, a subsample of patients with PD from two studies at the University Hospital of Cologne was used. For details on the objective and recruitment procedure, please refer to Ruppert et al. [27] and Hammes et al. [28]. Both studies were approved by the ethics committee of the medical faculty of the University of Cologne, Germany (approval numbers 12–265 and 15–325). Every subject provided informed consent to participate in the study. The authors assert that all procedures contributing to this work comply with the ethical standards of the relevant national and institutional committees on human experimentation and with the Helsinki Declaration of 1975, as revised in 2008.

For the current analyses, inclusion criteria were (i) the diagnosis of PD according to UK brain bank criteria; (ii) the availability of FDG-PET imaging data; (iii) the availability of neuropsychological data including the modified and extended version of the Subjective Memory Impairment Questionnaire, the SCD-Q [29, 30]; (iv) the exclusion of PD-MCI or PD dementia according to level-II diagnostic criteria;, and (v) the exclusion of other diseases affecting brain functions, e.g., operationalized by the intake of any centrally active medication such as antidepressant/ anxiolytics beyond the antiparkinsonian dopaminergic medication (which itself had to be stable for the last four weeks prior to and throughout study participation). These inclusion criteria were met by 30 patients (for details, see Flow Chart in Supplementary Material Fig. 1).

### Clinical aspects

Clinical characteristics such as disease duration and the levodopa equivalent daily dose [LEDD; 31] were recorded. Motor symptom severity was measured with the Unified Parkinson’s Disease Rating Scale part 3 [UPDRS-III; 32] in ON- and OFF-state [27]. Depressive symptoms were examined using the Beck Depression Inventory 2 [BDI-II; 33].

### Neuropsychological assessment

The neuropsychological assessment took place in the medication ON-state within 2 days of the FDG-PET scan.

#### Subjective cognitive decline

To assess SCD, a modified and extended version of the SCD-Q [29, 30] was used to cover a broader spectrum of cognitive functions that can be affected in PD. It assesses SCD over six cognitive domains: memory, attention, language, executive functions, visuo-cognitive skills, and social cognition. First, the patients have to answer a dichotomous question (“yes or no”) on whether they experience worsening in the respective domain. Second, if present, concerns and worries regarding this worsening are assessed on a 3-point Likert-scale (“no” = 0, “sometimes” = 1, “yes” = 2). Third, for each domain (irrespective of the answer to the first global question), questions about experiencing cognitive decline in specific daily situations have to be answered on a 4-point Likert-scale from “never” = 0 to “always” = 3. Summarizing, three scores can be created: (i) subjectively impaired cognitive domains (SCD-D, 0–6 points), (ii) presence of worries regarding the subjectively impaired cognitive domains (SCD-W, 0–12 points), and (iii) specific cognitive concerns in daily life situations (SCD-S, 0–60 points).

To assess SCD-related concepts and to validate our concept of SCD, additional questionnaires were used. The first additional measure was the informant-rated version of the Everyday Cognition Questionnaire (ECog) containing 39 items [34]. For each item, the informant has to compare the patient’s current state with the state 10 years prior and indicate whether the variable is unchanged or has worsened on a four-point-scale. The items are related to memory, language, visuo-cognitive functions, planning, organization, and attention. For evaluation, the sum score (maximum of 156 points) is divided by the number of items (maximum of 39 items) resulting in a score between 1 and 4 [34]. The second measure was the Cognitive Failures Questionnaire (CFQ) that is designed as self- and informant-reported questionnaire [35]. The CFQ contains 25 items indicating the frequency with which people experience cognitive failures in the past 6 months on a five-point-scale. Thus, maximum score is 100 points with lower scores indicating a lower frequency of cognitive failures [35]. Thirdly, the Dysexecutive Questionnaire (DEX), a subtest from the Behavioral Assessment of the Dysexecutive Syndrome, was used [36]. The DEX was designed to assess possible behavioral changes as a result of a dysexecutive syndrome. Both the self- and informant-rated version were used containing 20 items each that are rated in terms of frequency on a five-point-scale resulting in a total score ranging from 0 to 80, with lower scores indicating less problems with executive functioning [36].

For group comparisons in the present study, the presence of SCD is defined as experienced worsening in at least two domains accompanied by at least partially (“sometimes”) present worries. Following these criteria, *n* = 12 patients were classified as having no SCD (PD control group; PD-CG) and *n* = 18 as having SCD (PD-SCD). For the investigation of the relationship between SCD and SCD-related concepts, neural correlates of SCD, and other determinants of SCD, however, we used the SCD-S as a continuous measure within the analyses.

#### Cognition

As cognitive screening tools, the Mini-Mental State Examination [MMSE; 37] and the Parkinson Neuropsychometric Dementia Assessment [PANDA; 38] were used. To assess specific cognitive domains, additional neuropsychological tests were applied; that were also used to exclude the presence of objective cognitive impairment (PD-MCI or PD dementia). For the operationalization of Level-II diagnostic criteria for PD-MCI [39], the cut-off for impaired test performance was set at ≤  − 1.5 standard deviations according to published normative data. If two or more tests were impaired across the five assessed cognitive domains, the patient was excluded from the present analyses due to present objective cognitive impairment that might confound the SCD assessments. The cognitive screening tools were not relevant for excluding the presence of objective cognitive impairment. For further details on neuropsychological tests and their assignment to a cognitive domain, see Supplementary Material Table 1.

### FDG-PET imaging data acquisition and pre-processing

FDG-PET was performed under standard conditions with an average dose of 185 MBq FDG on a 24-detector ring high-resolution research tomograph (ECAT HRRT, Siemens) and preprocessed in SPM12 (http://www.fil.ion.ucl.ac.uk/spm/software/spm12) running on MATLAB R2018a (The MathWorks, Inc.). Image acquisition procedure and preprocessing have previously been described in detail [27, 40]. FDG-PET acquisition was conducted in the medication OFF-state after at least 12-h discontinuation of levodopa, amantadine and MAO-inhibitors, and 72-h withdrawal from dopamine agonists. For the current analysis, normalized image dimensions were 91/109/91 voxels (x/y/z) sized 2 × 2 × 2 mm.

### Statistical analyses

Statistical analyses were performed using SPSS 26 for Mac unless indicated otherwise. All data were tested for normal distribution with Shapiro–Wilk tests and for homogeneity of variances with Levene-tests. Comparisons of demographic, clinical, and cognitive variables between PD-CG and PD-SCD were calculated using independent sample *t* tests, Mann–Whitney *U* tests, or χ^2^ tests as appropriate. As effect sizes, Cohen’s *d* is reported for *t* tests and *r* for Mann–Whitney *U* tests, indicating small (*d* ≥ 0.2; *r* ≥ 0.1), moderate (*d* ≥ 0.5; *r* ≥ 0.3), or strong (*d* ≥ 0.8; *r* ≥ 0.5) effects. Spearman correlations were computed between the SCD-Q subscores (SCD-D, SCD-W, SCD-S) and the scores of questionnaires assessing related concepts, i.e., ECog, CFQ, and DEX.

FDG-PET correlates of SCD were analyzed in SPM12 by whole-brain voxel-wise regression with the SCD-S subscale of the SCD-Q, using age, sex, UPDRS-III OFF-state, and BDI-II scores as covariates, to assess the correlations between the specific concerns of SCD and FDG-metabolism in patients with PD. We chose the regression approach over a group comparison as we aimed to assess SCD as a continuous measure and no established criteria to categorize patients to PD-CG and PD-SCD were available. The initial threshold was set at *p* ≤ 0.01 with a cluster extent threshold of 100 voxels. Results were considered significant at *p* ≤ 0.05 uncorrected on cluster level. Although this is a less-conservative criterion, it is commonly used in studies investigating neural correlates of SCD and/or MCI in PD [9, 15, 26]. For anatomical identification of the local peaks, the WFU-PickAtlas Tool 2.4 (http://fmri.wfubmc.edu/software/pickatlas) was used to transform the coordinates to regions of the Automated Anatomical Labeling atlas (AAL).

In addition, the mean FDG uptake value in a region of interest (ROI) including fronto-medial, temporo-medial, and parieto-occipital areas was extracted using MarsBaR (http://marsbar.sourceforge.net). The ROI was defined based on previous literature on neural correlates of SCD in PD and AD. For specific regions included in the ROI, see Supplementary Material Table 2. The metabolism value of this ROI as well as age, sex, education, UPDRS-III ON-state, BDI-II, and PANDA were integrated into stepwise regression analyses to evaluate possible determinants of SCD in PD patients. Note, that we used UPDRS-III in OFF-state as covariate in the whole-brain voxel-wise regression analysis and in ON-state as possible determinant in the stepwise regression analysis. With this differentiation, the medication state corresponded between the UPDRS-III assessment and the FDG-PET acquisition and neuropsychological testing, respectively.

## Results

### Sample characteristics

Sociodemographic and clinical characteristics as well as the overall cognitive state of the patients are displayed in Table 1. On average, patients were 66.90 ± 7.20 years old with a median PD duration of 3.5 years. PD-CG (*n* = 12) and PD-SCD (*n* = 18) did not differ significantly in sociodemographic variables, clinical characteristics, their global cognitive status, and further cognitive assessments.

**Table 1:** Sample characteristics

		Total (*n* = 30)		PD-CG (*n* = 12)		PD-SCD (*n* = 18)		Test statistic	*p* value	
Age		66.90	(7.20)	68.00	(5.59)	66.17	(8.17)	*t* = 0.68	.504	
Sex	Female, *n* (%)	7 (23.3)		4 (33.3)		3 (16.7)		χ^2^ = 0.12	.392	
	Male, *n* (%)	23 (76.7)		8 (67.7)		15 (83.3)				
**Education** in years, median (range)		14.50	(10–18)	15.00	(10–18)	13.50	(10–18)	*U* = 95.5	.602	
**Disease duration** in years, median (range)		3.50	(1–13)	3.00	(1–11)	4.00	(1–13)	*U* = 83.5	.305	
**UPDRS III — OFF**		25.73	(10.12)	24.25	(9.10)	26.72	(10.89)	*t* = − 0.65	.522	
**UPDRS III — ON**		19.83	(9.29)	19.75	(9.16)	19.89	(9.65)	*t* = − 0.40	.968	
**LEDD** in mg		403.26	(210.35)	399.79	(251.59)	405.57	(185.76)	*t* = − 0.72	.943	
MMSE, median (range)		29.00	(23–30)	29.00	(28–30)	29.00	(23–30)	*U* = 105	.917	
PANDA		24.17	(4.04)	23.83	(3.56)	24.39	(4.42)	*t* = 0.36	.719	
**Depressive symptoms** BDI-II		9.10	(5.68)	8.00	(4.11)	9.83	(6.54)	*t* =- 0.86	.396	
SCD-D, median (range)		3.00	(0–6)	1.00	(0–3)	5.00	(2–6)	*U* = 4	** ≤ .001**	***
SCD-W, median (range)		2.50	(0–12)	0.5	(0-2)	5.00	(1–12)	*U* = 4.5	** ≤ .001**	***
SCD-S		11.57	(6.87)	6.08	(3.87)	15.22	(5.95)	*t* = − 4.70	** ≤ .001**	***
ECog, median (range)		1.36	(1.00–2.36)	1.18	(1.00–1.72)	1.57	(1.00–2.36)	*U* = 23	** ≤ .001**	***
CFQ	Self-rated	28.70	(11.39)	21.75	(8.86)	33.33	(10.66)	*t* = − 3.11	**.040**	*
	Informant-rated, median (range)	22.00	(0–45)	19.00	(0–26)	25.00	(5–45)	*U* = 52.5	.053	
DEX	Self-rated	17.87	(11.43)	10.58	(5.68)	22.72	(11.81)	*t* = - 3.30	**.003**	**
	Informant-rated	14.43	(9.40)	11.64	(5.99)	16.24	(10.85)	*t* = − 1.28	.212	
Wechsler Memory Scale										
Digit span forward		48.87	(10.53)	50.67	(11.98)	47.67	(9.62)	*t* = 0.76	.545	
Digit span backward, median (range)		47.00	(34–71)	49.00	(37–71)	47.00	(34–71)	*U* = 104.5	.884	
Block span forward		46.87	(9.96)	47.17	(7.85)	46.67	(11.37)	*t* = 0.13	.896	
Block span backward, median (range)		47.00	(37–71)	47.00	(37–63)	45.50	(37–71)	*U* = 83.5	.305	
**Boston Naming Test**, median (range)		15.00	(14–15)	15.00	(14–15)	15.00	(14–15)	*U* = 27.5	.227	
Regensburger Wortflüssigkeitstest										
B words		54.27	(9.74)	54.50	(9.31)	54.11	(10.28)	*t* = 0.11	.917	
S words		52.97	(8.96)	53.00	(9.39)	52.94	(8.94)	*t* = 0.02	.986	
Alternating G-R words, median (range)		50.00	(40–74)	49.00	(40–62)	50.00	(43–74)	*U* = 95.5	.777	
Animals		54.45	(8.53)	55.83	(8.90)	53.47	(8.40)	*t* = 0.73	.473	
Jobs		55.07	(8.62)	56.42	(7.73)	54.12	(9.31)	*t* = 0.70	.489	
Alternating categories sport-fruit, median (range)		55.00	(29–69)	57.00	(39–69)	53.00	(29–63)	*U* = 68.5	.140	
Wisconsin Card Sorting Test										
Errors		44.89	(3.81)	44.75	(4.16)	45.00	(3.67)	*t* = − 0.17	.867	

*Note*. Data are presented as mean (standard deviation) unless indicated otherwise. For group comparisons, *p* values of independent sample *t* tests, Mann–Whitney *U* tests, or Chi-square tests are reported as appropriate. Variables were previously inspected by Shapiro–Wilk tests for normal distribution. *BDI-II* Beck Depression Inventory 2; *CG* control group; *CFQ* Cognitive Failures Questionnaire; *DEX* Dysexecutive Questionnaire; *ECog* Everyday Cognition Questionnaire; *PD* Parkinson’s disease; *MMSE* Mini-Mental State Examination; *PANDA* Parkinson Neuropsychometric Dementia Assessment; *SCD* subjective cognitive decline; *SCD-D*, subjective cognitive decline-questionnaire domains; *SCD-S* subjective cognitive decline-questionnaire specific concerns; *SCD-W* subjective cognitive decline-questionnaire worries; *UPDRS-III* Unified Parkinson Disease Rating Scale part 3. *p ≤ .05; **p ≤ .01; ***p ≤ .001

### Subjective cognitive decline (SCD)

In comparison to PD-CG, PD-SCD descriptively showed higher scores in all subscores of the SCD-Q and questionnaires assessing SCD related constructs (Table 1). The differences reached significance for all subscores of the SCD-Q (SCD-D: *U* = 4.00, *p* < 0.001, *r* = 0.82; SCD-W: *U* = 4.50, *p* < 0.001, *r* = 0.81; SCD-S: *t*(28) =  − 4.69, *p* < 0.001, *d* = 1.75), the ECog (*U* = 23.00, *p* < 0.001, *r* =  − 0.62), and the self-rated version of both CFQ (*t*(28) =  − 3.11, *p* = 0.04, *d* = 1.16) and DEX (*t*(28) =  − 3.30, *p* = 0.003, *d* = 1.23).

Spearman correlations between the SCD-Q subscores and further SCD related variables are reported in Table 2. As only the subscore SCD-S was further used in the FDG-PET analysis and the stepwise regression analysis, here we exemplary outline further details of the correlations for the SCD-S subscore. The SCD-S correlated strongly and highly significantly with the self-rated version of the CFQ (*r* = 0.59, *p* = 0.001) and the self-rated version of the DEX (*r* = 0.62, *p* < 0.001). Weak but significant correlations were observed with the ECog (*r* = 0.44, *p* = 0.018). Correlations between SCD-S and the informant-rated versions of both CFQ (*r* = 0.28, *p* = 0.157) and DEX (*r* = 0.10, *p* = 0.615) did not reach statistical significance.

**Table 2:**
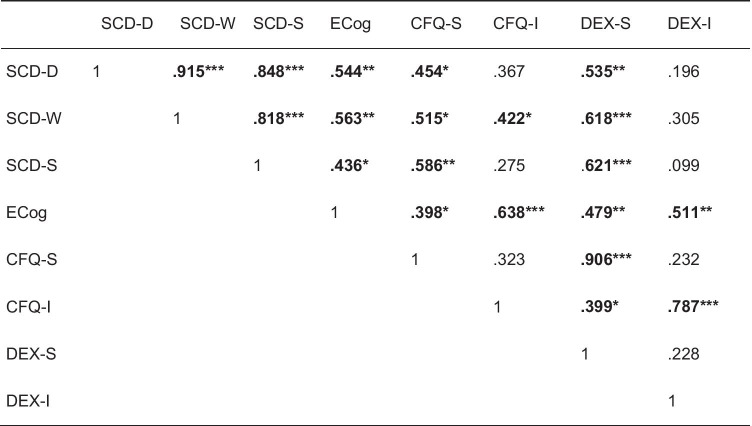
Spearman correlations between subjective cognitive decline and related concepts

*Note*. *CFQ-I* Cognitive Failures Questionnaire informant-rated version; *CFQ-S* Cognitive Failures Questionnaire self-rated version; *DEX-I* Dysexecutive Questionnaire informant-rated version; *DEX-S* Dysexecutive Questionnaire self-rated version; *ECog* Everyday Cognition Questionnaire; *SCD-D* subjective cognitive decline-questionnaire domains; *SCD-S* subjective cognitive decline-questionnaire specific concerns; *SCD-W* subjective cognitive decline-questionnaire worries

^*^p ≤ .05; **p ≤ .01; ***p ≤ .001

### Neural correlates of SCD

**Fig. 1:**
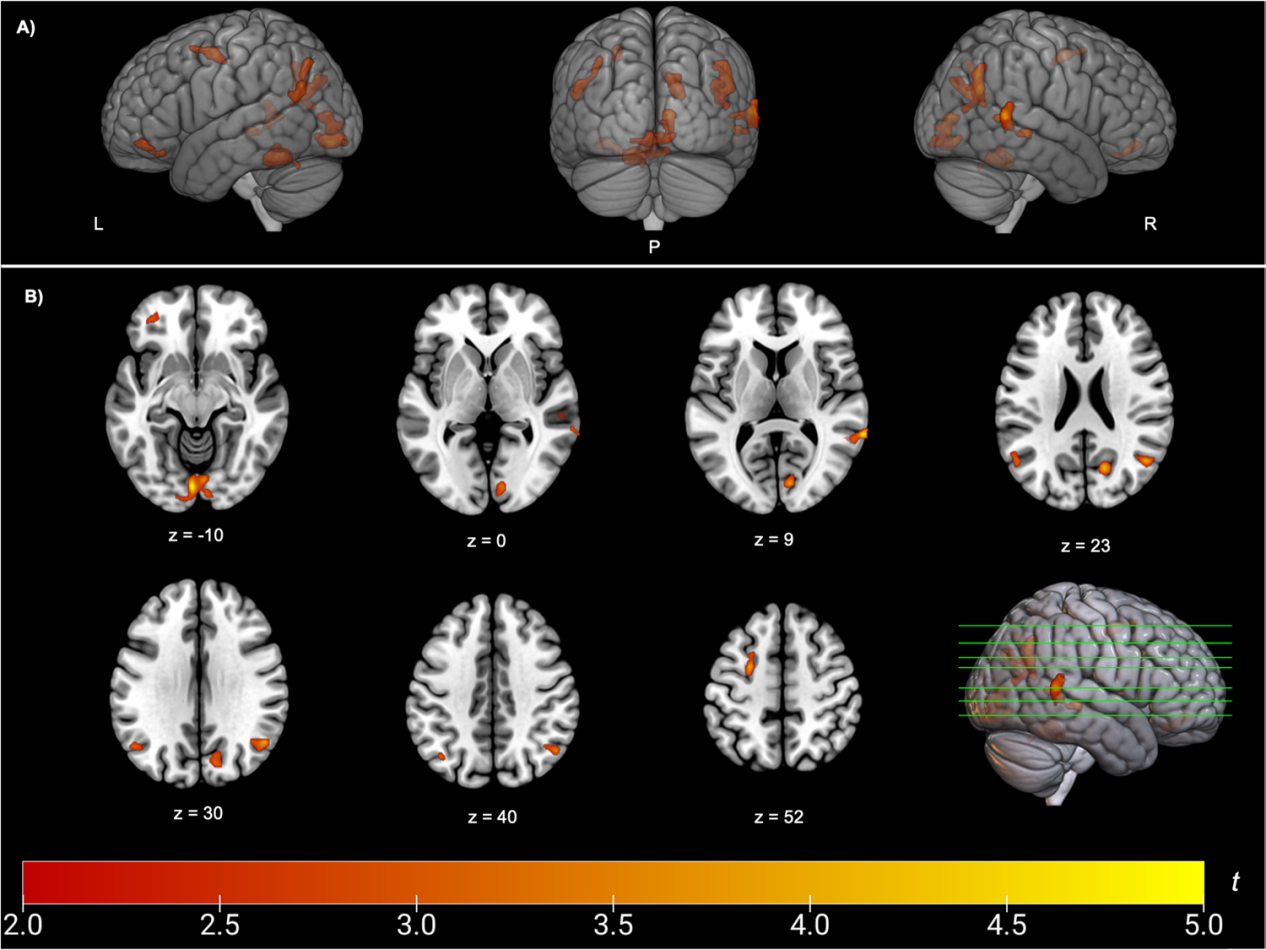
Whole brain voxel-wise regression analysis between FDG uptake and SCD-S score. Red-yellow maps illustrate negative correlation between FDG uptake and SCD-S score indicating hypometabolism in these areas. **A** 3D render view **B** 2D axial slices. L, left; P, posterior; R, right. Color bar indicates *t* values. Slices and 3D display were constructed with MRIcroGL, neurological display

The whole brain voxel-wise regression analysis between FDG-metabolism and SCD-S score is presented in Table 3 and visualized in Fig. 1. Patients with higher SCD-S scores showed decreased metabolism in the following regions: right angular gyrus (*p* = 0.002, *k*_e_ = 1223), bilateral middle temporal gyrus (left: *p* = 0.026, *k*_e_ = 570; right: *p* = 0.009, *k*_e_ = 841), bilateral occipital regions including right precuneus (*p* = 0.012, *k*_e_ = 766), and left calcarine sulcus (*p* = 0.001, *k*_e_ = 1594), left middle frontal gyrus (*p* = 0.044, *k*_e_ = 448) including the orbital part (*p* = 0.043, *k*_e_ = 455). Higher SCD-S scores did not correlate with increased metabolism in any region.

**Table 3:** Whole brain voxel-wise regression analysis

Peak coordinates (MNI)			Anatomical region	Side	Cluster level				
X	Y	Z			FDR-corr	uncorr		cluster size	*T*
− 26	− 1	52	Middle frontal gyrus	L	.737	.044	*	448	3.93
− 24	8	53	Superior frontal gyrus	L	-	-		-	3.41
− 27	39	− 15	Middle frontal gyrus, orbital part	L	.737	.043	*	455	4.42
− 22	32	− 19	Middle frontal gyrus	L	-	-		-	3.21
67	− 45	9	Middle temporal gyrus	R	.290	.009	**	841	4.88
57	− 30	− 4	Middle temporal gyrus	R	-	-		-	4.39
59	− 49	7	Superior temporal gyrus	R	-	-		-	3.83
− 54	− 60	20	Middle temporal gyrus	L	.571	.026	*	570	3.54
− 38	− 69	45	Inferior parietal lobule	L	-	-		-	3.29
− 41	− 66	37	Inferior parietal lobule	L	-	-		-	3.19
50	− 62	26	Angular gyrus	R	.948	.002	**	1223	4.76
50	− 63	− 36	Tuber (Cerebellum)	R	-	-		-	4.10
49	− 72	14	Middle temporal gyrus	R	-	-		-	3.14
− 4	− 85	− 10	Calcarine sulcus	L	.060	.001	***	1594	5.27
11	− 83	7	Cuneus	R	-	-		-	4.31
5	− 79	-8	Lingual gyrus	R	-	-		-	4.15
16	− 67	23	Precuneus	R	.309	.012	*	766	4.13
12	− 78	31	Cuneus	R	-	-		-	3.27

*Note.* SCD-S was entered as dependent variable. Age, sex, UPDRS-III OFF-state, and BDI-II were used as covariates. *FDR-corr.* False Discovery Rate Correction; *L* left; *MNI* Montreal Neurological Institute; *R* right

^*^p ≤ .05; **p ≤ .01; ***p ≤ .001

### Predictors of SCD

Stepwise regression analyses with age, sex, education, UPDRS-III ON-state, BDI-II, PANDA, and the ROI value as predictors were conducted to identify predictors of SCD, indicated by the subscore SCD-S. The final model explained 53.7% (adjusted *R*^*2*^ = 50.3%) of the variance in the SCD-S score with only two significant predictors: depressive symptoms and ROI metabolism value (for details, see Table 4). Increasing depressive symptoms as measured with the BDI-II (*β* = 0.467, *p* = 0.002) correlated with more SCD, whereas a decreased FDG-metabolism in the above-mentioned ROIs was related to more SCD (*β* =  − 0.442, *p* = 0.003).

**Table 4:** Final model of the stepwise regression analysis

Predictor	*B*	SE	β	*t*	*p*	
Final model						
BDI-II	.564	.166	.467	3.399	**.002**	**
ROI	− .834	.259	− .442	− 3.222	**.003**	**
F(2, 27) = 15.667, *p* < 0.001; *R*^*2*^ = .537; adjusted *R*^*2*^ = .503						
Excluded variables						
Age			.140	1.067	.296	
Education			− .018	− 0.127	.900	
Sex			− .154	− 1.158	.257	
PANDA			− .189	− 1.430	.165	
UPDRS-III ON			.196	1.524	.140	

*Note*. SCD-S (specific concerns) subscore was used as dependent variable in the regression model. Sex was dummy-coded with 0 = male, 1 = female. *BDI-II* Beck Depression Inventory 2; *PANDA* Parkinson Neuropsychometric Dementia Assessment; *UPDRS-III* Unified Parkinson Disease Rating Scale part 3; *ROI* region of interest

^*^p ≤ 0.05; **p ≤ 0.01; ***p ≤ 0.001

## Discussion

This study on demographic and behavioral determinants as well as on neural correlates of SCD in patients with PD revealed (i) decreased levels of FDG-metabolism in middle frontal, middle temporal and occipital areas, as well as in the angular gyrus in patients with higher SCD-S scores, and (ii) higher levels of depressive symptoms and a reduced regional FDG-metabolism in fronto-medial, temporo-medial, and parieto-occipital regions to be predictive of SCD.

One strength of this study is the focus on the broader SCD construct targeting a spectrum of cognitive domains instead of restricted concepts like SCD in the memory domain. This recognizes that cognitive decline in PD is highly heterogeneous and not focused on memory impairment only [11–14]. So far, no extensively validated or commonly used tools to assess SCD exist [7], which is why we assessed relationships of the SCD-Q subscales with other questionnaires. Importantly, correlational analyses revealed an association of the subscore SCD-S that was further used in the analyses and self-rated questionnaires assessing SCD-related concepts. However, no correlation was found between SCD-S and informant-rated versions of the SCD-related questionnaires. This is congruent with the definition of SCD as a self-perceived phenomenon [1]. Furthermore, patients with and without SCD showed different levels of SCD and SCD-related concepts in the expected direction, with the PD-SCD group reporting higher levels of subjective complaints than the control group across different assessments. Importantly, no significant differences were found in objective cognitive functioning, clinical, or sociodemographic variables between those two groups. Additionally, the exclusion of patients with objective cognitive deficits in our study cohort was based on the established level-II diagnostic criteria for PD-MCI [39] and precluded the confounding of SCD by objective cognitive deficits.

Our findings on functional neuroimaging correlates of SCD are congruent with prior neuroimaging studies on SCD in memory in PD [9, 15, 26] and AD [22]. Especially the middle frontal and middle temporal areas are involved in executive functions and semantic processing [11, 12]. In combination with parieto-occipital areas, processing visuo-cognitive functions, these structures are also part of the attentional system [11, 12]. Therefore, the spectrum of neural correlates might reflect a pre-clinical stage of cognitive impairment in PD, which already mirrors the heterogeneity of objective cognitive impairment in PD, for example, as stated in the dual syndrome hypothesis [11, 12]. Further supporting this hypothesis, Huang et al. [41, 42] found FDG-hypometabolism in the middle frontal gyrus and inferior parietal lobule in patients with PD-MCI, indicating that the pattern of hypometabolism we detected in PD-SCD might represent early PD-related pathological changes. Glucose hypometabolism was also found in parieto-occipital areas of the brain in patients with PD and objective cognitive dysfunctions [43]. Importantly, it has been shown previously that metabolic reductions in occipital brain areas herald the onset of PDD [44]. Furthermore, parieto-occipital hypometabolism is a prominent feature to discriminate Lewy-Body-dementia and PDD from AD [45, 46]. Overall, the broad spectrum of neuronal correlates of SCD in PD highlights the importance of assessing SCD as a broad construct not limited to perceived memory impairment.

Regional FDG-hypometabolism in fronto-medial, temporo-medial, and parieto-occipital regions was also found to significantly predict SCD in PD within our stepwise regression analyses, which additionally investigated the influence of demographic, clinical, and neuropsychological predictors of SCD in PD. Next to FDG-metabolism, depressive symptoms emerged as an independent significant positive predictor of SCD in PD. Depressive symptoms have been identified as a reliable determinant of SCD in the general aging context before [16, 19, 20]. As Burmester et al. [16] discuss in their systematic review, depressive symptoms might even account for any observed relationship between objective cognitive functions and SCD. This in turn might explain the finding of global cognitive functioning assessed with the PANDA not being a significant predictor for SCD in our PD cohort. However, it can also be hypothesized that the phenomenon of depressive realism might account for the strong relationship of depressive symptoms and SCD, as the depressive symptomatology might lead to excessively negative or “skeptical” perceptions of one’s cognitive state. In this context, the PANDA cognitive screening tool was probably not sensitive enough to reflect any associations of SCD with objective cognitive performance. Furthermore, it was previously shown that the relationship between subjective and objective cognitive performance is rather unreliable anyhow [47].

Demographic variables such as age, education, and sex were not identified as significant independent determinants of SCD in our cohort. In the general aging context, SCD were found to be more frequent in women than men and positively related to age and education [16]. In this context, however, for example, the role of age-related stereotypes and the perception of one’s own health are discussed. In PD patients, general age-related stereotypes might be less important than perceived stereotypes associated with PD itself. However, PD motor impairment as assessed with the UPDRS-III did not emerge as an independent significant determinant of SCD either. Motor impairment was previously found to be a significant predictor for objective cognitive decline in PD, arguing that more severe motor impairment might indicate more severe neuro-pathological changes that in turn increase the risk for cognitive deficits [48]. However, our sample of patients with PD had no objective cognitive impairment and motor impairment was only mild. Therefore, the extent to which inferences from motor impairment to SCD were drawn was probably reduced. This hypothesis is strengthened by findings of the only study investigating determinants of SCD in the memory domain in PD so far [21]. In this study, motor impairment was a significant predictor of SCD in the memory domain in patients with PD-MCI, but not for patients without objective cognitive impairment, which is in agreement with our results.

One limitation is that the sample size was relatively small. However, our findings are supported by previous literature, which in turn supports our approach to measure SCD and implies that it is sensitive enough to detect effects in a relatively small sample. The major strength of this study, as already discussed above, was the operationalization of the SCD construct based on sound diagnostic criteria excluding objective cognitive impairment by level-II diagnostic criteria of PD-MCI [39] and using an elaborated SCD questionnaire assessing several cognitive domains and daily life situations. Furthermore, to the author’s best knowledge, this analysis constitutes the first approach of combining research on neuronal and sociodemographic, clinical, and neuropsychological correlates of SCD in PD.

Summarizing, the present study strengthens that SCD may be an early manifestation of future cognitive decline and, more generally speaking, early pathological changes in PD. Objective, validated screening tools and diagnostic criteria for SCD in PD should be developed. We identified hypometabolism in fronto-medial, temporo-medial, and parieto-occipital regions as well as more depressive symptoms as independent determinants of SCD in PD. The early identification of vulnerability for future cognitive decline constitutes the basis for successful prevention and delaying the onset of objective cognitive decline. Furthermore, the better understanding of determinants of SCD might help to develop targeted treatment approaches in cognitive prevention, training, and rehabilitation.

## Supplementary Information

Below is the link to the electronic supplementary material.

Supplementary file1 (PDF 52 KB) The Supplementary Material for this article includes one Figure visualizing the study flow, and two Tables. Table 1 of the Supplementary Material gives an overview of the neuropsychological test battery used for the exclusion of PD-MCI, and Table 2 provides a list of the AAL regions implemented in the ROI for which the metabolism value was extracted.

## Data Availability

The datasets generated during and/or analyzed during the current study are available from the corresponding author on reasonable request.

## Code availability

Code used to analyze the data of the current study is available from the corresponding author on reasonable request.
