# Supplementary material for: Neural correlates and predictors of subjective cognitive decline in patients with Parkinson’s disease
Source: Neurol Sci. 2021 Nov 25;43(5):3153–63. doi: 10.1007/s10072-021-05734-w (PMC9018636; doi:10.1007/s10072-021-05734-w)
Supplement: Supplementary file 1 — Supplementary file1 (PDF 52 KB) The Supplementary Material for this article includes one Figure visualizing the study flow, and two Tables. Table 1 of the Supplementary Material gives an overview of the neuropsychological test battery used for the exclusion of PD-MCI, and Table 2 provides a list of the AAL regions implemented in the ROI for which the metabolism value was extracted. [file 10072_2021_5734_MOESM1_ESM.pdf]

Supplementary Material

NEURAL CORRELATES AND PREDICTORS OF SUBJECTIVE COGNITIVE DECLINE IN PATIENTS WITH PARKINSON'S DISEASE

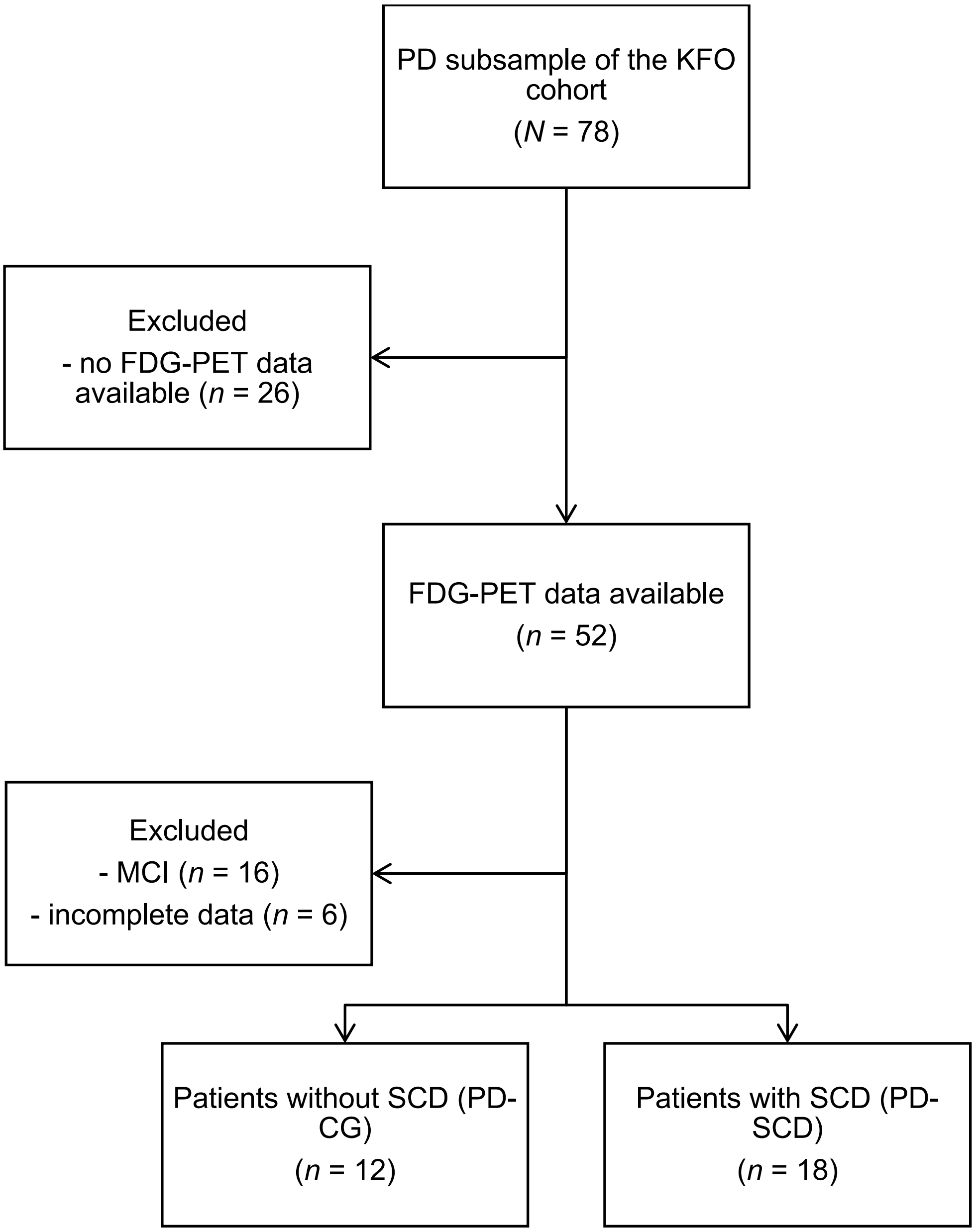

Figure 1. *Study flow.*
CG, control group; KFO, Klinische Forschungsgruppe (engl. clinical research unit); MCI, mild cognitive impairment; PD, Parkinson's Disease; SCD, subjective cognitive decline

Table 1. *Neuropsychological tests for cognitive assessment.*

Domain	Test/Subscale		Maximum Score
**Memory**	PANDA	Word pair learning	5
	PANDA	Word pair delayed recall	7
	MMSE	Word list recall	3
**Attention**	PANDA	Digit span	6
	WMS	Digit span forward	12
	WMS	Digit span backward	12
**Executive functions**	RWT	B-words	-
	RWT	S-words	-
	RWT	Alternating G-R-words	-
	RWT	Alternating categories: sport - fruit	-
	WCST	Categories	-
	WCST	Errors	-
	PANDA	Alternating categories: animal - furniture	7
**Language**	RWT	Animals	-
	RWT	Jobs	-
	BNT		15
	MMSE	Language task	9
**Visuospatial skills**	WMS	Block span forward	12
	WMS	Block span backward	12
	MMSE	Pentagons	1
	PANDA	Spatial imagery task	5

Note. For the operationalization of Level-II diagnostic criteria for PD-MCI, the cut-off for impaired test performance was set at ≤-1.5 SD according to published normative data, if available. However, we also had to include subtests of the cognitive screening tools MMSE and PANDA to assess each cognitive domain with at least two tests. As no normative data for these screening subtests were available, the values of 30 healthy controls from the KFO-cohort and another ongoing study at the Department of Neurology from the University Hospital of Cologne, addressing impaired self-awareness in patients with PD-MCI, were used. *z*-scores were derived by subtracting the mean score of those controls from our patients' score, divided by the standard deviation of the control cohort.
BNT, Boston Naming Test, Kaplan, Goodglass, and Weintraub (1983); MMSE, Mini-Mental State Examination, Folstein, Folstein, and McHugh (1975); PANDA, Parkinson Neuropsychometric Dementia Assessment, Kalbe et al. (2008); RWT, Regensburger Wortflüssigkeitstest (Regensburg verbal fluency task), Aschenbrenner, Tucha, and Lange (2000); WMS, Wechsler Memory Scale, Molz and Schulze (2010); WCST, Wisconsin Card Sorting Test, Nelson (1976).

Table 2. *AAL regions implemented in the ROI.*

AAL region	Combined ROI
5. Frontal_Sup_Orb_L	X
6. Frontal_Sup_Orb_R	X
9. Frontal_Mid_Orb_L	X
10. Frontal_Mid_Orb_R	X
23. Frontal_Sup_Medial_L	X
24. Frontal_Sup_Medial_R	X
25. Frontal_Med_Orb_L	X
26. Frontal_Med_Orb_R	X
31. Cingulum_Ant_L	X
32. Cingulum_Ant_R	X
37. Hippocampus_L	X
38. Hippocampus_R	X
39. ParaHippocampal_L	X
40. ParaHippocampal_R	X
43. Calcarine_L	X
44. Calcarine_R	X
45. Cuneus_L	X
46. Cuneus_R	X
47. Lingual_L	X
48. Lingual_R	X
59. Parietal_Sup_L	X
60. Parietal_Sup_R	X
61. Parietal_Inf_L	X
62. Parietal_Inf_R	X
63. SupraMarginal_L	X
64. SupraMarginal_R	X
65. Angular_L	X
66. Angular_R	X
67. Precuneus_L	X
68. Precuneus_R	X
81. Temporal_Sup_L	X
82. Temporal_Sup_R	X
85. Temporal_Mid_L	X
86. Temporal_Mid_R	X
89. Temporal_Inf_L	X
90. Temporal_Inf_R	X

Note. AAL=Automated Anatomical Labeling; ROI=Region of Interest.

References

Aschenbrenner, S., Tucha, O., & Lange, K. (2000). Regensburger Wortflüssigkeitstest Hogrefe Göttingen. In: Germany.

Folstein, M. F., Folstein, S. E., & McHugh, P. R. (1975). “Mini-mental state”: a practical method for grading the cognitive state of patients for the clinician. *Journal of Psychiatric Research, 12*(3), 189-198.

Kalbe, E., Calabrese, P., Kohn, N., Hilker, R., Riedel, O., Wittchen, H.-U., . . . Kessler, J. (2008). Screening for cognitive deficits in Parkinson's disease with the Parkinson neuropsychometric dementia assessment (PANDA) instrument. *Parkinsonism & Related Disorders, 14*(2), 93-101.

Kaplan, E., Goodglass, H., & Weintraub, S. (1983). The Boston Naming Test. Lea & Febiger. *Philadelphia, PA*.

Molz, C., & Schulze, R. (2010). Wechsler Intelligenztest für Erwachsene WIE. Deutschsprachige Bearbeitung und Adaptation des WAIS-III von David Wechsler. *Report Psychologie, Jg, 36*, 369-371.

Nelson, H. E. (1976). A modified card sorting test sensitive to frontal lobe defects. *Cortex, 12*(4), 313-324.
